# Primary Ventricular Fibrillation in a Patient with Mild Hypercalcemia

**DOI:** 10.5935/abc.20180059

**Published:** 2018-04

**Authors:** Rita Marinheiro, Leonor Parreira, Pedro Amador, Francisco Sardinha, Sara Gonçalves, Sónia Serra

**Affiliations:** Centro Hospitalar de Setúbal, Setúbal, Lisboa - Portugal

**Keywords:** Ventricular Fibrillation, Shock, Cardiogenic, Hypercalcemia, Syncope, Unconsciousness

## Introduction

An abnormally short QT interval can be caused by several situations such as hypercalcemia, hyperkalemia, acidosis, hyperthermia, effects of drugs like digitalis or congenital short QT syndrome (SQTS).^1^ Primary hyperparathyroidism (PHPT) can ultimately cause short QT interval since overproduction of parathyroid hormone (PTH) causes hypercalcemia. However, cardiac arrhythmias are uncommon and electrical storm has been rarely described in patients with hypercalcemia.^2^

Secondary causes of short QT must be ruled out before considering the diagnosis of SQTS.^3^ First described in 2000, SQTS is a congenital primary electric disorder characterized by abnormally short corrected QT interval (QTc) on the surface electrocardiogram (ECG) that is associated with sudden cardiac death (SCD) in individuals with structurally normal heart. According to 2015 ESC Guidelines for the management of patients with ventricular arrhythmias,^4^ SQTS is diagnosed in the presence of a QTc ≤ 330 msec or it can be diagnosed in the presence of a QTc < 360 ms and one or more of the following factors: pathogenic mutation, family history of SQTS, family history of sudden death before 40 years old and/or survival of a ventricular tacchycardia (VT)/ ventricular fibrillation (VF) episode in the absence of heart disease.

The authors present a case of an electrical storm due to polymorphic VT suspected to be caused by SQTS. However, PHPT was diagnosed one year later and mild hypercalcemia was thought to have been the cause or a contributor for the electrical storm.

## Case Report

**Figure 1:**
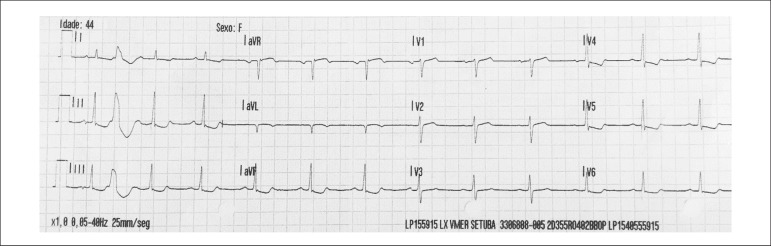
Twelve-lead electrocardiogram (ECG) taken by emergency team before hospital admission demonstrating a corrected QT interval (according to Bazett´s formula) of 349 milliseconds (msec).

**Figure 2:**
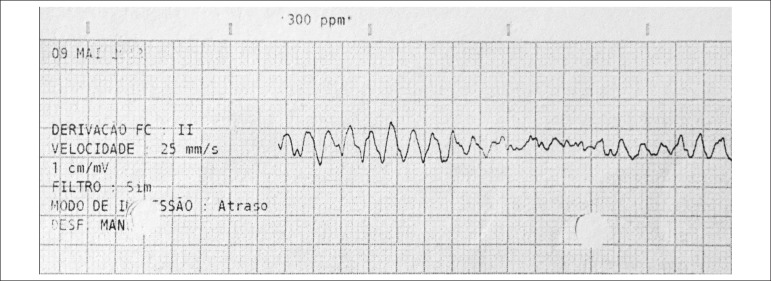
Electrocardiogram (ECG) during ventricular fibrillation (VF) episode.

A previous healthy 44-year-old woman was brought to the emergency room after an unwitnessed fall followed by extreme anxiety. She had no respiratory distress or others symptoms; she denied cardiovascular risk factors or alcohol and drugs consumption. Her family history was irrelevant. On physical examination, she was calm, apyretic, hemodynamically stable and her neurological exam and cardiac and pulmonary auscultations were normal. Her blood pressure was 90/61 mmHg and heart rate (HR) was 114 beats per minute (bpm). While she was under observation, she *lost consciousness.* Cardiac monitoring confirmed a VF episode and the patient was shocked and recovered. Her laboratory values were normal, including hemogram, electrolytes, renal function, thyroid hormones, cardiac enzymes and serum D-dimer. Her total calcium was 9.3 mg/dL and albumin was 3.0 g/dl (normal range 3.5 - 5.0 g/dL). The corrected calcium for hypoalbuminemia was 10.3 mg/dL (normal range 8.4 - 10.2 mg/dL). She had an ECG taken by emergency team before hospital admission (Figure 1) that showed a sinus rhythm at a HR of 75 bpm, normal PR interval (160ms) and QRS duration (90 bpm), no ST changes and a QTc of 349ms (according to Bazett´s formula). T_peak_ - T_end_ interval (0.50 msec) and T_peak_- T_end_ / QT ratio (0.18) were not prolonged. Short QT interval was not detected in the subsequent ECGs, including in the one performed after the first shock. In the next hours, cardiac monitoring demonstrated premature ventricular contractions (PVC) with distinct morphologies and R-on-T phenomenon, which was responsible for polymorphic VT that degenerated to VF (Figure 2). Ten external shocks were applied and treatment with amiodarone and beta-blockers was ineffective. Sedation and orotracheal intubation were decided due to the requirement of successive shocks. Emergency coronary angiography excluded coronary artery disease (CAD). Since paroxysmal VT were presumably caused by PVC with short coupling intervals (“R-on-T” extrasystoles falling on the peak of the T wave), isoproterenol infusion was started (0.08 mg/h). HR increased and arrhythmic episodes disappeared. Twenty-four hours later this treatment was stopped and no more arrhythmias were detected.

A comprehensive approach was performed. During hospitalization, successive ECGs did not show short QTc interval or other alterations. PVCs were noted in some instances on ECGs but they had different morphologies and only a few of them had a short coupling interval. Laboratory values remain normal. Transthoracic echocardiogram was normal and cardiac magnetic resonance imaging (MRI) did not visualize late enhancement or other changes. Flecainide test was negative and electrophysiologic study (EPS) was normal with no arrhythmia induction. The treadmill test (Bruce protocol) was performed. In rest, her QT interval was 320 ms and HR 90 bpm (QTc = 392 ms); in peak effort (HR = 134 bpm), QT was 280ms (QTc = 418 ms). She requested to terminate the test in stage 1 (1.7 mph at 10% grade) since she was very tired.

Further investigation for SQTS as the cause of VF was not performed and a single chamber implantable cardioverter-defibrillator (ICD) (ProtectaVR D364VRM, MedtronicÒ) was implanted for secondary prevention. The patient was discharged with no medical therapy. Fifteen days later, the patient complained of palpitations and the ICD interrogation demonstrated non-sustained VT initiated by PVC with short coupling intervals. Quinidine was initiated and symptoms as well as non-sustained VT episodes disappeared.

After one year, a laboratory study was done once again due to complains of asthenia and anorexia. Serum calcium was 10.2 mg/dL and albumin 3.2 g/dL, corrected calcium was 10,8 mg/dL. Serum phosphorus was 1.8 mg/dL (normal range 2,7 - 4,5 mg/dL). Potassium and magnesium were normal. Based on these results, PTH measurement was performed and it was elevated: 344.8 pg/mL (normal range 15 - 68.3 pg/mL) and PHPT was diagnosed. Bone densitometry (DEXA), renal function and urine calcium were normal. The patient was referred to endocrinology surgery but according to the NIH *criteria* for ***parathyroidectomy, the surgery was not recommended.*** She remains asymptomatic with no further VT episodes or frequent PVCs. 

## Discussion

VF causes vary according to the age group. In young, it is mostly due to channelopathies, cardiomyopathies, myocarditis and substance abuse, while in patients older than 40 years, CAD is the leading cause.^4^ Taking into account the patient´s age, it seemed reasonable to perform coronary angiography. Brugada´s pattern was not evident but regarding intermittent alterations in this syndrome and the good response to isoproterenol, a flecainide test was performed to exclude this diagnosis. Cardiac MRI was also crucial to exclude cardiomyopathy. Although the EPS is not indicated to stratify risk in SQTS since its sensitivity and negative predictive value are low,^5^ the SQTS diagnosis was not absolutely certain and so the EPS was performed and it was normal. Since SQTS patients show a reduced adaptation of the QT interval to HR,^6^ the patient underwent the treadmill test but she did not reach maximum predicted HR. However, the variation from rest to peak effort of 40 ms is probably attenuated. After excluding all other causes of electric storm, SQTS was considered a reasonable diagnosis based on absence of structural heart disease, normal laboratory values and the presence of a short QT interval in one ECG. Serum calcium was only slightly increased (10,3 mg/dL) so secondary causes of SQTS were considered to be absent. According to the ESC guidelines, a SQTS diagnosis can be made based on a QTc < 360 ms and an episode of VF without structural heart disease.^4^ The absence of short QT in the subsequent ECGs as well as the absence of other common electrocardiographic features present in SQTS (short ST segment and prolonged T_peak_ - T_end_ interval and T_peak_ - T_end_ / QT ratio),^7^ make SQTS diagnosis less probable. It is unclear if short QT interval can be intermittent or whether fluctuating QT intervals are of clinical significance in patients with SQTS.^8^ Of note, a case of sudden cardiac death associated with intermittent short QT interval has been described.^9^ Mazzanti et al.^10^ proposed that SQTS and Brugada Syndrome (BrS) may have some features in common and intermittent pattern of short QT interval (same as ST elevation in right precordial leads) seems reasonable. The presence of short action potential duration, as well as abbreviated repolarization, suggests that the R-on-T phenomenon may precipitate arrhythmogenesis in SQTS. Obviously, performing genetic testing could be considered. Five genes have been linked to SQTS (KCNH2, KCNQ1, KCNJ2, CACNA1C and CACNB2b), but the yield of genetic screening remains low (20% overall).^10^ In other words, the chances of a gene mutation be identified and confirm the diagnosis is low and a negative test does not rule out SQTS since there are mutations unidentified. Besides, our patient had no offspring or siblings so it was considered that genetic test would not add relevant information or change therapeutic management. The good response to quinidine in the follow-up supports the diagnosis of SQTS since quinidine can reduce arrhythmic events in this entity.^5^

 The authors admit that alternative diagnosis can be considered. The occurrence of malignant ventricular arrhythmias in patients with PVCs with short coupling interval has been extensively reported. In these cases, PVCs have the same morphology suggesting one focal origin. Left bundle branch morphology and left axis were identified as most commonly related to VF,^11^ which is usually not induced by an EP study. Verapamil is reported to be effective in suppressing these arrhythmias, while quinidine, b-blockers and amiodarone are ineffective. In our patient, quinidine is effective, PVCs had distinct morphologies and initially PVCs were suppressed by isoproterenol, which is not a consistent finding in these cases. Of note, transient metabolic or electrolytic disorders can influence PVC susceptibility to degenerate in VF^12^ so hypercalcemia could have contributed to this phenomenon.

*The initial diagnosis was rethought several months later when PHPT was confirmed although* it is not clear if the arrhythmic events can be caused by mild hypercalcemia. Other reported cases described more severe hypercalcemia associated with arrhythmias. Alternatively, mild hypercalcemia could have been a trigger to ventricular arrhythmias in the case of SQTS or PVCs with short coupling. In fact, the patient had higher levels of calcium while on therapy with quinidine and no arrhythmias occurred. To establish a cause-effect relationship it is necessary to demonstrate that calcium perfusion would cause VF in EPS as described by Chang et al.^11^ However it would imply repeating EPS with calcium perfusion and facing a potential electrical storm which could be difficult to control as it had been in the first episode. For these reasons, the authors considered it inappropriate.

## Conclusion

The authors report a case of electrical storm possibly related to SQTS taking into account the presence of short QT interval and isoproterenol and quinidine efficacy.^13^ However, it is not clear why short QT interval was present only in the first ECG and secondary causes could not be completely ruled out since mild hypercalcemia was present.

Up until now there are no reports regarding mild hypercalcemia as a cause of arrhythmic storm. The final diagnosis is still not certain but EPS with calcium perfusion could be dangerous and genetic testing yield in SQTS is too low to justify its use. Although without a definitive diagnosis, the authors emphasize the importance of excluding all reversible causes, especially in case of subtle hydroelectrolytic disorders like the one presented above.
